# Long delay in diagnosis of a case with MEN1 due to concomitant presence of AIMAH with insulinoma: a case report and literature review

**DOI:** 10.1186/s12902-022-01022-6

**Published:** 2022-04-21

**Authors:** Vajihe Chavoshi, Seyed Saeed Tamehri Zadeh, Shayesteh Khalili, Amirhassan Rabbani, Seyed Amir Hassan Matini, Zhaleh Mohsenifar, Farzad Hadaegh

**Affiliations:** 1 Prevention of Metabolic Disorders Research Center, Research Institute for Endocrine Sciences, Shahid Beheshti University of Medical Sciences, No. 24, Parvaneh Street, Velenjak, Tehran, Iran; 2 Department of Internal Medicine, School of Medicine, Imam Hossein Hospital, Shahid Beheshti University of Medical Sciences, Tehran, Iran; 3 Department of Transplant & Hepatobiliary Surgery, Taleghani Hospital, Shahid Beheshti University of Medical Sciences, Tehran, Iran; 4 4Department of Pathology, Kashan University of Medical Sciences, Kashan, Iran; 5 Taleghani General Hospital, SBMU, Tehran, Iran

**Keywords:** MEN-1, AIMAH, Insulinoma, Genetic mutation, Case report

## Abstract

**Background:**

ACTH-independent macronodular hyperplasia (AIMAH) is an uncommon disorder characterized by massive enlargement of both adrenal glands and hypersecretion of cortisol. Concomitant AIMAH and multiple endocrine neoplasia type1 (MEN1) is rare to our knowledge.

**Case presentation:**

Herein, we describe a 32 year old woman with long history of prolactinoma and secondary ammonhrea presented with not-severe manifestation of hypoglycemia due to concomitant presence of insulinoma with AIMAH leading to 12 years delay of MEN1 diagnosis. Laboratory tests showed severe hypoglycemia associated with hyper insulinemia (non-fasting blood sugar = 43 mg/dl, insulin = 80.6 μIU /ml, C-peptide = 9.3 ng/ml) hyperparathyroidism (calcium = 10.3 mg/dl, phosphor = 3.1 mg/dl, PTH = 280 pg/ml) and chemical evidence of an ACTH-independent hypercortisolism (serum cortisol value of 3.5, after 1 mg dexamethasone suppression test serum ACTH value of 17 pg/ml, and high urinary cortisol level). Abdominal CT scan demonstrated two enhancing well-defined masses 27*20 mm and 37*30 mm in the tail and body of the pancreas, respectively, and a 36*15 mm mass in left adrenal gland (seven Hounsfield units). Dynamic pituitary MRI revealed a partial empty sella. The physical examination of the patient was unremarkable. Distal pancreatectomy and a left adrenalectomy were performed. After the surgery, we observed clinical and biochemical remission of hyper insulinemia and gradual decrease in urinary cortisol. The histological features of the removed left adrenal gland were consistent with AIMAH. Histological examination of the pancreatic lesions revealed well differentiated neuroendocrine tumors. Genetic abnormalities in the *MEN1,* heterozygote for pathogenic variant chr11; 645,773,330-64577333AGAC, c.249-252delGTCT, p. (11e85Serfs Ter33) in exon 2 were found. It was recommended the patient undergoes parathyroidectomy as soon as possible.

**Conclusion:**

Given the history and presentation of our case, we recommend that the clinicians consider the possibility of autonomous cortisol production in MEN1 patients who do not show severe symptoms of hypoglycemia in the presence of insulinoma.

## Background

ACTH- independent macronodular adrenal hyperplasia (AIMAH) is a rare disorder characterized by enormous bilateral enlargement of adrenal glands with hypercortisolism [1, 2]. AIMAH comprises of approximately 1% of ACTH-independent Cushing syndrome (CS) cases, and the majority of them present with overt CS [3]. Most AIMAH cases are sporadic, and only few such cases with a positive family history have been reported. Although familial cases showed an autosomal dominant inheritance pattern, the genetic mutation has not been identified [4, 5].

Multiple endocrine neoplasia type1 (MEN1), a rare autosomal dominant syndrome, impacts multiple endocrine organs, primarily including parathyroid, anterior pituitary, and pancreas [6]. Adrenal lesions can be detected in 30%—40% of MEN1 patients. Adrenal lesions in MEN1 patients encompass different subtypes from benign adenomas to adrenocortical carcinomas. It has been observed that a significant proportion of adrenal lesions are without any clinical symptoms and functional AIMAH is extremely uncommon. Pancreatic lesions are common in patients with MEN1 and are the main cause of mortality related to the disease [7]. Gastrinoma is the most common pancreatic lesion in MEN-1 patients; however, insulinoma is more common in young patients [8]. We present a case of MEN1 syndrome concomitant with AIMAH that probably masked the severe hypoglycemic manifestations of insulinoma, leading to a 12- year delay of diagnosis.

## Case presentation

A 32-year-old woman was referred to the endocrinology clinic for further assessment of recurrent severe hypoglycemia. Menarche occurred at 15 years of age. She reported secondary amenorrhea accompanied by headache and spontaneous galactorrhea from the age of 20. Diagnosis of prolactinoma was confirmed based on high blood prolactin level, and MRI of the pituitary gland and 0.5 mg cabergoline, a dopamine agonist, twice a week was initiated. She became pregnant at age 27 using ovulation induction by Clomiphene, and gave birth to a healthy baby without any complications. Two years after delivery, due to persistent amenorrhea and galactorrhea, a pituitary MRI was performed and showed a large (21*17*14 mm) mass with a leftward deviation of the pituitary stalk. Optic chiasm was intact. During the past year, the patient had several admissions in ED following episodes of severe headache, fatigue, weakness which were reportedly relieved with intravenous dextrose (unfortunately, the data on electrolytes and glucose level were not available). There were no symptoms of hyperandrogenism, weight change, hypertension, and diarrhea.

**Fig. 1:**
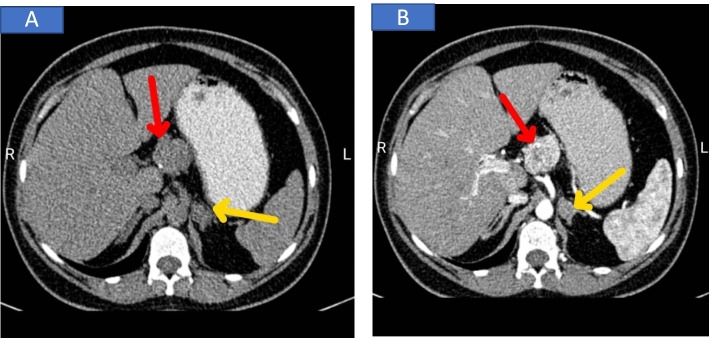
Without contrast (**a**) and with contrast (**b**), axial contrast-enhanced CT of the abdomen shows the hyperenhancing pancreatic body mass (red arrows) as well as indeterminate left adrenal nodules (yellow arrows)

**Fig. 2:**
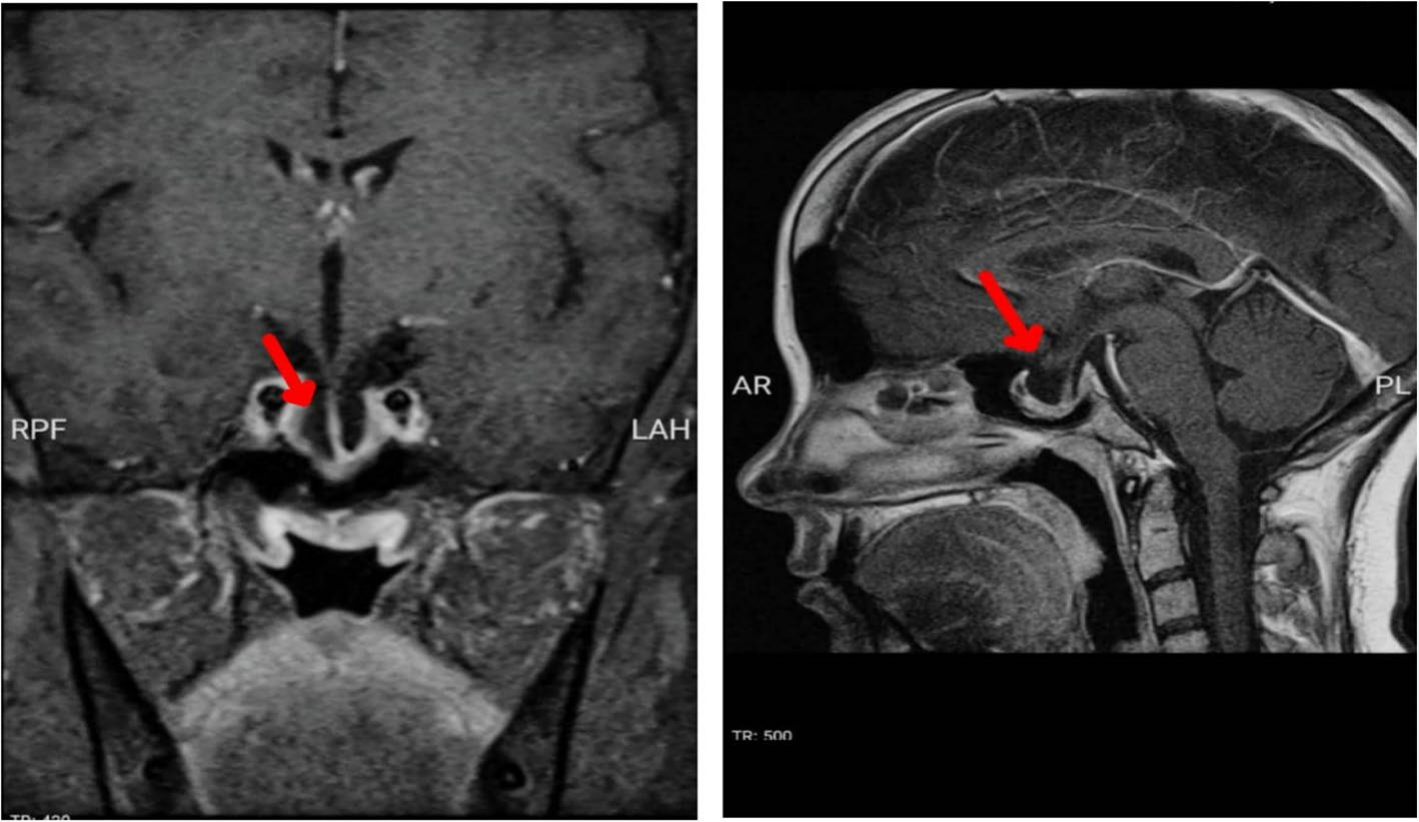
Pituitary MRI revlead partially empty sella

**Fig. 3:**
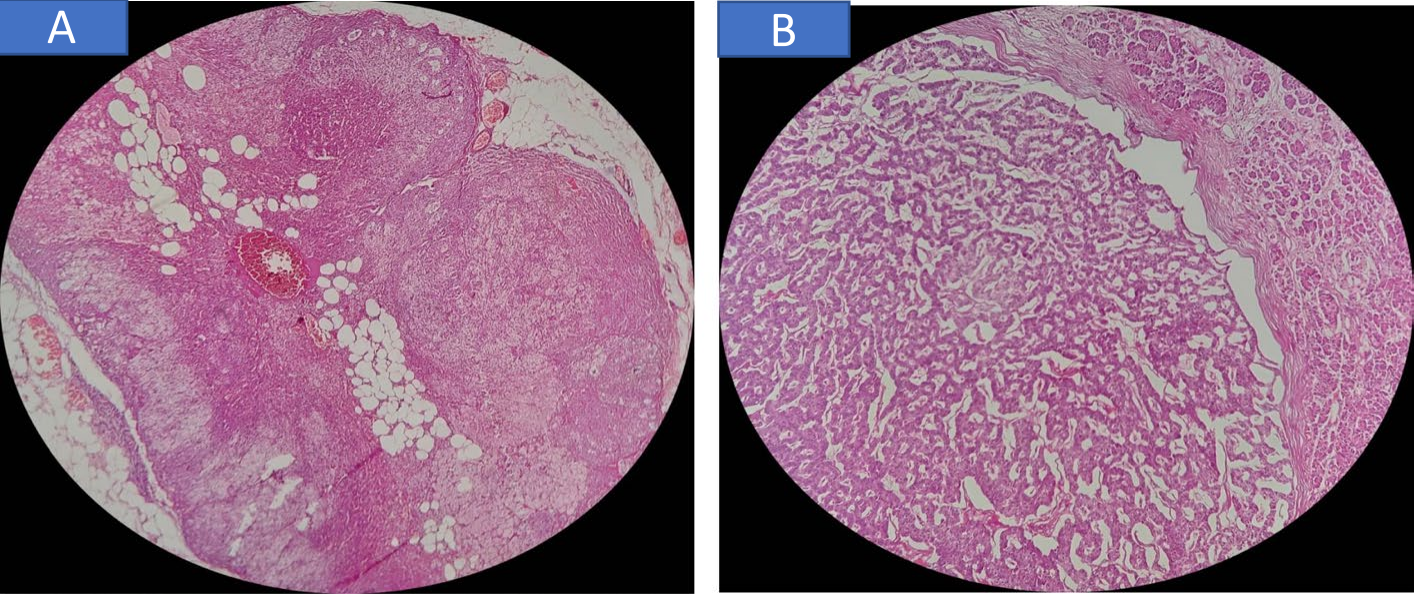
**A** Macronodular adrenal hyperplasia showing nodules composed of clear and compact cells with variable lipid (H&E,X400, Nikon microscope, Eclipse E100) (**B**) Normal pancreas( right side) and lesion ( left side) (H&E, X100, Nikon microscope, Eclipse E100)

The physical examination of the patient was unremarkable. With suspicion to insulinoma and MEN-1, several blood tests were ordered, which are presented in Table 1. Decreased level of blood sugar and increased level of insulin and C-peptide indicated insulinoma. A raised calcium and PTH level suggested primary hyperparathyroidism Abdominal ultrasound and CT scan were also performed. Ultrasonography revealed two hypoechoic mass-like lesions measuring 27*20 and 10*7 mm near together adjacent to the tail of pancreas, and spiral abdominal CT scan disclosed two enhancing well-defined masses 27*20 mm and 37*30 mm in the tail and body of the pancreases, respectively, calcified areas in larger mass, and a 36*15 mm mass in the left adrenal gland (seven Hounsfield units) (Fig. 1). Results of the blood and urine examinations on second admission are presented in Table 2. By considering MEN1, plasma cortisol after overnight dexamethasone suppression test and urinary free cortisol were requested, which were increased. Also, ACTH level was measured which wasdecreased, indicating primary hypercortisolism. Dynamic pituitary MRI was revealed a partial empty sella (Fig. 2). Bone mineral density (BMD) was performed, which is presented in Table 3. Endoscopic ultrasound (EUS) showed 21*22 mm hypoechoic round lesion with well-defined border in pancreatic body, it was hyper vascular in Doppler and its elastic ratio was 7. Fine needle aspiration (FNA) using EUS from the pancreas masses was performed and an expert pathologist reported neoplastic process with neuroendocrine feature with KI-67 < 2%. Hence, in the patient with the diagnosis of MEN-1 with components of primary hyperparathyroidism, prolactinoma, insulinoma, and CS due to possible adrenal adenoma a spleen-preserving distal pancreatectomy and a left adrenalectomy was performed by a hepatobiliary surgeon. The whole left adrenal gland was enormously enlarged, measuring 100 × 60 × 20 mm. On sectioning, an adrenal tissue with nodular appearance was observed, the maximum of the nodule reached to 60 × 10 mm, which indicated macronodular hyperplasia (H&E,X400, Nikon microscope, Eclipse E100) |(Fig. 3A). In addition, two nodular masses 30 × 30 mm and 20 × 20 mm in the head and tail of pancreas were detected, respectively (H&E, X100, Nikon microscope, Eclipse E100) (Fig. 3B). Tumors were confined to pancreas and all margins were free of tumor. Lymphovascular and perineural invasion were not identified and the tumors stages were T2 NO M_x_. After transient hyperglycemia during four days following the surgery that managed with insulin, the level of BS was returned to within reference range. The results of the cortisol and urine free cortisol following the surgery are shown in Table 4. Considering the high level of prolactin at the time of discharge from the hospital (466 ng/ml), low bone mass, the presence of empty sella, and the long duration of amenorrhea, the patient was recommended for 1 mg per week Cabergoline and hormone replacement therapy. Finally, parathyroidectomy was considered for the patients as well.

**Table 1:** Blood examination on first admission

Variables	value	Normal range
**cortisol 4PM (ug/dl)**	**7.7**	**6–18.4**
**Random blood sugar (mg/dl)**	**43**	**60–100**
**Insulin (μIU /ml)**	**80.6**	**2.6–24.9**
**C-peptide (ng/ml)**	**9.3**	**1.1–4.4**
**Prolactin (ng /pml)**	**20.2**	**1.9–25**
**TSH (μIU/mL)**	**1.52**	**0.3–5**
**FT4 (ng/dL)**	**5.31**	**0.67–1.5**
**calcium (mg/dl)**	**10.3**	**8.6–10.3**
**phosphorus ( mg/dl)**	**3.1**	**2.6–4.5**
**PTH (pg/ml)**	**280**	**15–65**
**ALP (ng/ml)**	**564**	**64–306**
**FSH (mIU/ml)**	**5**	**1–9**
**LH (mIU/ml)**	**5.8**	**1.68–15**

**Table 2:** Blood and urine examinations for assessing MEN-1

Variables	value	Normal range
cortisol 8AM (ug/dl)	**18.6**	**6–18.4**
ACTH (pg/ml)	**17**	**Up to 64**
24-h urine free cortisol (μg/24 h)	**281**	**50–190**
dexamethasone suppression test (ug/dl)	**3.5**	** < 5.0**
DHEAS (ug/dl)	**30.3**	**95.8–511.7**
gastrin (pg/ml)	**78.4**	**13–115**
urine 24-h metanephrine (μg/24 h)	**99**	** < 350**
normetanephrine (μg/24 h)	**299**	** < 600**
Vanyl mandelic acid (mg/24 h)	**5.1**	** < 13.6**
calcium (mg/dl)	**10.2**	**8.6–10.3**
phosphorus ( mg/dl)	**2.4**	**2.6–4.5**
PTH (pg/ml)	**323**	**15–65**
vitamin D (nmol/l)	**3**	**30–100**

**Table 3:** BMD of lumbar, spine, hip, and forearm on the HOLOGIC explorer QDR series DXA

yScan	BMD (g/cm^2^)	T-score	Fracture risk	Z-score
Lumbar spine	**0.757**	**-3.1**	**High**	**-3.2**
Total femur	**0.916**	**-0.2**	**Low**	**-0.2**
Femoral neck	**0.747**	**-0.9**	**Low**	**-0.8**
Forearm	**0.525**	**-2.8**	**High**	**-2.6**

**Table 4:** Trend of adrenal tests from 14 days before the surgery to 4 months after the surgery

Tests	Reference range	Two weeks before the surgery	One week before the surgery	One week after the surgery	Three weeks after the surgery	Two months after the surgery	Four months after the surgery
ACTH (pg/ml)	**Up to 64**	**15**	**17**		**9.9**		
Cortisol morning (µg/dl)	**6.2–19.4**	**7.7**	**18.6**	**26.7**	**16.4**		
24-h urine free cortisol (µg/dl)	**50–190**		**281**	**736**	**390.3**	**8.7**	**6.3**
Overnight dexamethasone suppression test (µg/dl)	** < 5.0**		**3.5**				**0.7**

DNA was extracted from blood cells using salting out method. PCR was used to amplify for 10 coding exons as well as 20 bases of flanking non-coding sequences of MEN1 gene [NM-000244.3]. After cleaning of the PCR products, cycle sequencing was carried out using the ABI Big Dye Terminator v.3.0 kit. Products were resolved by electrophoresis on an ABI 3130 capillary sequencer. Sequencing was performed separately in both the forward and reverse directions. Alignment followed by comparing the individual’s sequences with the reference sequences was performed, respectively. The results indicated that she is heterozygote for pathogenic variant chr11;645,773,330-64577333AGAC, c.249-252delGTCT, p. (11e85Serfs Ter33) in exon 2 of MEN1 gene. This variant has been reported as a pathogenic change in www.ensemble.org and www.ncbi.nlm.nih.gov/clinvar and OSMIC. The above variant has been previously observed in MEN1 patients [9, 10]. Bioinformatics prediction program (Mutation Taster) states that protein features might be affected due to this deletion. Also, according to the American College of Medical Genetics and Genomics (ACMG) standards and guidelines, this variant is classified as pathogenic [11].

There was no remarkable family history except her father had a history of renal stones and lithotripsy; however, his PTH and serum calcium were normal. MEN1 mutations were assessed for her 5-year-old daughter, and the result of the test came back positive (similar mutation to her mother).

## Search strategy for literature review

We carried out a data search of PubMed for articles published from January 1990 to February 2021 using the following keywords “MEN-1 and adrenal lesions”, “MEN-1 and AIAMAH”, MEN-1 and autonomous cortisol secretion”, “MEN-1 and cortisol secretion, and MEN-1 and macronodular hyperplasia”. All relevant studies were retrieved, and we also checked their references manually to find any other related papers.

## Discussion and conclusion

According to the literature review, corticoadrenal lesions can be detected in 30 to 40% of patients with MEN-1 [12]. A variety of adrenal abnormalities, mainly including non-malignant adenoma and hyperplasia, and less common carcinoma, in patients with MEN-1, can be identified, and the majority of these abnormalities are non-functional [12]. To the best of our knowledge, the concomitant presentation of MEN-1 and macronodular adrenal hyperplasia have been reported in only 11 cases (Table 5) and that four were labeled as AIMAH [12–15]. Of 4 AIMAH cases, three patients had parathyroid tumors [12, 13, 15], one patient had pituitary tumor (nonfunctional) [12], and two patients had pancreatic tumor (insulinoma) [12, 14].

**Table5:** Demographics, gland involvement, treatment, and genetic features of MEN1 patients with macronodular hyperplasia (current case)

Author, [reference]	year	Gender	Parathyroid lesion(pos/neg)	Pituitary Lesion (pos/neg)	Pancreas Lesion (pos/neg)	Treatment	Mutation
**Burgess **[16]	**1996**	**2 patients, 1 female and 1 male**	**Positive for both**	**Negative for 1, not described for the other**	**Positive for both, gastrinoma for 1, not described for the other**	**Right adrenalectomy for 1, the other patient died**	**Not performed**
**Barzon **[17]	**2001**	**2 patients, not described**	**Not clearly identified**	**Not clearly identified**	**Not clearly identified**	**Not clearly identified**	**Not clearly identified**
**Langer **[18]	**2002**	**2 patients, both female**	**Not described**	**Not described**	**Not described**	**Bilateral adrenalectomy for both patients**	**E191X for 1 patient, splice-site for the other patient**
**Waldmann **[19]	**2007**	**1 patient, female**	**Not described**	**Not described**	**Not described**	**Left adrenalectomy**	**L168P Ex 3**
**Sato **[15]	**2006**	**1 patient, female**	**positive**	**negative**	**negative**	**Left adrenalectomy**	**Not performed**
**Hsiao **[13]	**2009**	**1 patient, Male**	**positive**	**negative**	**negative**	**Not described**	**Pro494Leu**
**Lee **[14]	**2011**	**1 patient, female**	**negative**	**negative**	**Positive, insulinoma**	**Bilateral adrenalectomy, pancreas lesion was removed**	**Not performed**
**Yoshida **[12]	**2011**	**1 patient, male**	**positive**	**positive**	**Positive, insulinoma**	**Right adrenalectomy, spleen-preserving pancreatectomy**	**No mutation**
**Current case**	**2021**	**1 patient, female**	**positive**	**positive**	**Positive, insulinoma**	**left adrenalectomy, spleen-preserving distal pancreatectomy**	**11e85Serfs Ter33**

The most similar case to our patient was described by Yoshida et al. [12]. They described a patient with AIMAH, hyperparathyroidism, pituitary microadenoma, and multifocal insulinoma in addition to diabetes mellitus. All diagnostic tests for Cushing syndrome were normal other than high midnight cortisol concentrations which probably explain insulin resistance and diabetes mellitus. It is noteworthy to mention that our patient, despite repetitive hypoglycemic presentation with weakness, did not report other neuroglycopenic symptoms such as seizure or altered mental status. We assume that autonomous cortisol production by AIMAH can attenuate the hypoglycemic manifestations due to insulin resistance.

In the study of Skogseid et al. [20] and Burgess et al. [16], all MEN-1 patients with adrenal lesions had pancreatic neuroendocrine tumors, similar to our case diagnosed with insulinoma. In addition, it has shown that hyperinsulinemia had some role in adrenocortical tumors development, which probably can be from heterogeneous nature of adrenal glands in proliferation and function [21]. Since the presence of adrenal tumor in sporadic insulinoma is not common, this hypothesis merits further investigations. [17]. It is important to mention that considering the close correlation between neuroendocrine pancreatic tumors and adrenal lesions in MEN 1, abnormality in the genome may be behind this association. Another hypothesis is that hypoglycemia due to hyperinsulinemia, stimulates the secretion of hormones, such as catecholamine and ACTH, which may lead to the proliferation of adrenal cells. However, this theory is unlikely to explain adrenal hyperplasia in patients with MEN-1, because according to Yoshida’s report [12], adrenal gland growth was continuing after the removal of insulinoma.

Mutations in MEN-1 gene, which is located in 11q13 chromosome, are the main cause of MEN-1 in the majority of the patients, even though it has been demonstrated that those mutations are not found in 10% to 30% of the patients. In addition to MEN-1 mutations, in some cases, gross deletions, p27, and p18 defects may contribute to MEN-1 [9]. In the study of Hsiao et al., among 16 patients with AIMAH, MEN-1 mutation (Pro494Leu) was detected in only one patient [13]. Yoshida et al. found no mutations in MEN-1, p27, and p18 genes [22]. Langer et al. pointed out that MEN1 mutations in exon 2 are expected to be observed more than other mutations. They found MEN1 mutation in exon 2 in out of two patients with macronodular hyperplasia [18]. In the present study, the patient was positive for MEN1 mutation in exon 2 as well.

As we mentioned before, AIMAH is characterized by huge bilateral adrenal enlargement; however, up to now, similar to our case, three cases of unilateral AIMAH has been reported [23–25]; therefore, the diagnosis of AIMAH cannot be ruled out based on the absence of bilateral lesions at imaging as it has been emphasized that the diagnosis of AIMAH is mostly based on pathological examinations [26]. Thus, in our case, we cannot rule out absence of nodular changes in right adrenal and the right adrenal involvement may be less prominent than left adrenal to be observed at imaging. Also*,* according to findings of Sheikh-Ahmad et al., the probability of achieving CS remission of people with AIMAH after unilateral adrenalectomy was 94.4% for years [27]; thus, it is necessary to follow our patient for CS recurrence**.**

The progression of AIMAH is extremely slow, and it takes years to lose the diurnal plasma ACTH rhythm. In AIMAH, plasma ACTH is intended to be decrease and would not be fully suppressed, especially in those with mild cortisol hypersecretion. Similarly, in our patient, ACTH did not fully suppress. The other reasons can be offered ectopic ACTH production by the adrenal glands [28] or in those with GR loss-of-function mutations [29].

A number of literature proposed bilateral laparoscopic adrenalectomy as the optimal treatment for bilateral AIMAH [30, 31]. A study claimed no death attributed to surgery was found in 45 patients with bilateral AIMAH experiencing bilateral adrenalectomy [31]. Unilateral adrenalectomy due to no need for life-tong steroid replacement was suggested by several studies [32, 33]. In a study with a median of 69 months follow-up, the success rate of unilateral adrenalectomy was estimated to be approximately 93% [33]. Likewise, we performed unilateral adrenalectomy (left-sided) plus distal pancreatectomy for our patient, and after the surgery, we observed a constant reduction in UFC.

Although a number of studies have been dedicated to clarify the mechanism of AIMAH, up to now, the precise mechanism is not elucidated. Considering the relevant studies, the pathogenesis of AIMAH can be heterogeneous [1, 34, 35]. While clonal studies reveal that polyclonal pattern can be involved in AIMAH genesis [35], other studies demonstrate that the pathogenesis behind the disease is related to cAMP/protein kinase A pathway [36]. Furthermore, it has been hypothesized that prolonged stimulation of aberrant receptors expressed within the adrenal cortex through several ligands may explain this adrenal abnormality [1, 2].

In the current study, we describe the case of MEN1 with simultaneous presentation of insulinoma and AIMAH that was demonstrated by hormonal and pathological evaluations. Considering the history of our case, we suggest clinicians think of autonomous cortisol production in MEN1 patients who do not show severe symptoms of hypoglycemia in the presence of insulinoma (s).

## Data Availability

All data used during the current study are available from the corresponding author on reasonable request.

## Abbreviations

AIMAH: ACTH-independent macronodular adrenal hyperplasia
MEN1: Multiple Endocrine Neoplasia MEN1
CS: Cushing Syndrome
ACTH: Adrenocorticotrophic, Hormone
LH: Luteinizing Hormone
FSH: Follicle-stimulating Hormone
PTH: Parathyroid Hormone
TSH: Thyroid-stimulating Hormone
DHEAS: Dehydroepiandrosterone Sulfate
ED: Emergency Department
MRI: Magnetic Resonance Imaging
CT scan: Computed Tomography scan
EUS: Endoscopic Ultrasound
FNA: Fine Needle Aspiration
